# Protective action of N-acetylcysteine on sperm quality in cyclophosphamide-induced testicular toxicity in male Wistar rats

**DOI:** 10.5935/1518-0557.20180079

**Published:** 2019

**Authors:** Seyyid A Shittu, Shehu-Tijani Shittu, Opeyemi O Akindele, Olufadekemi T Kunle-Alabi, Yinusa Raji

**Affiliations:** 1 Laboratory for Reproductive Physiology and Developmental Programming, Department of Physiology, College of Medicine, University of Ibadan, Ibadan, Nigeria; 2 Endocrinology and Metabolism Unit, Department of Physiology, College of Medicine, University of Ibadan, Ibadan, Nigeria

**Keywords:** N-acetylcysteine, adjunct drug, sperm quality, chemotherapy

## Abstract

**Background:**

Reductions in sperm quality due to free radical formation during cancer chemotherapy are well documented, hence the need for an adjunct antioxidant treatment during chemotherapy. This study was designed to investigate the effects of N-acetylcysteine on sperm quality following cyclophosphamide exposure in male Wistar rats.

**Methods:**

Twenty male Wistar rats weighing 150-170g were randomly assigned into 4 groups of five rats each, and were orally administered distilled water (Control), Cyclophosphamide (6mg/kg), N-acetylcysteine (100mg/kg) or Cyclophosphamide + N-acetylcysteine for 21 days. Sperm count, histone-protamine replacement, chromatin integrity, testicular histomorphometry and BAX Protein expression were assessed using standard procedures. The data was presented as mean ± SEM and analyzed using students' t- test. A *p*<0.05 was considered significant.

**Results:**

Sperm counts were significantly reduced (*p*<0.05) among the cyclophosphamide (69.95±7.78 x10^6^/ml) and cyclophosphamide + N-acetylcysteine (64.78±3.52 x10^6^/ml) treated rats, while it increased significantly (*p*<0.05) in the N-acetylcysteine (132.20±28.71 x10^6^/ml) treated rats compared to the control animals (115.30±8.70x10^6^/ml). Increased interstitial space distance, degenerated Leydig cells and impaired histone-protamine replacement observed among the cyclophosphamide-treated rats were ameliorated in the cyclophosphamide + N-acetylcysteine-treated rats. Sperm chromatin integrity, which was poor in the cyclophosphamide-treated rats, was considerably improved when compared with the Control and the N-acetylcysteine-treated rats. Bax protein expression was reduced in the cyclophosphamide (20%) and cyclophosphamide+N-acetylcysteine (20%) groups when compared with the Control (50%) and N-acetylcysteine (50%) groups.

**Conclusion:**

We concluded that N-acetylcysteine might improve sperm histone protamine replacement, which is one of the stage-specific effect of cyclophosphamide toxicity.

## INTRODUCTION

Spermatogenesis is a series of events involving the production of spermatozoa with a characteristic genomic compaction, capable of surviving different environments within the parent organism until fertilization (Montellier *et al.,* 2013). The events are vulnerable to the buildup of errors (Georgiou *et al*., 2006); thus, sperm must be correctly programmed and packaged to successfully pass on genetic and epigenetic information to the developing embryo. To enhance these processes, sperm undergoes some changes in the chromatin structure by replacing histones within the cell chromatin in a larger percentage with cysteine-rich protamine during spermiogenesis, a process known as protamination (Oliva, 2006). In addition, formation of disulfides through DNA cross-linkages further condense the chromatin, thereby stabilizing the compacted sperm DNA (Calvin & Bedford, 1971). However, abnormal deposition of sperm protamines during spermiogenesis or altered chromatin condensation can lead to enhanced susceptibility to sperm DNA injury (Kosower *et al.*, 1992; Lolis *et al.*, 1996; Codrington *et al*., 2004). Analysis of possible alterations to DNA integrity has been suggested to be a more objective and better prognostic marker for fertility potential of spermatozoa because it provides evidence of hidden anomalies that might exist even in spermatozoa appearing morphologically normal (Bianchi *et al.*, 1996; Evenson *et al.*, 1999; 2002). Reports have also shown that incorrect DNA integrity and condensation due to failure during spermiogenesis (histone to protamine exchange) seems to be an important factor predicting the outcome of assisted reproduction (Blanchard *et al*., 1990; Ankem *et al*., 2002; Steger *et al*., 2003).

Exposure to toxic alkylating agents, such as some chemotherapeutic agents, among which is cyclophosphamide (Spermon *et al*., 2006; Codrington *et al*., 2007), may significantly contribute to the impairment of chromatin compaction, thus increasing the susceptibility of these cells to oxidative or apoptotic attack (Sega, 1990). Cyclophosphamide is widely used to treat malignant and non-malignant tumors (Alyamkina *et al*., 2010). However, like other chemotherapeutic agents, it produces some side effects such as severe cytotoxicity, hemorrhagic cystitis, and temporary infertility (Wang *et al*., 2012; Alkam *et al*., 2014).

N-Acetylcysteine (NAC) is an antioxidant and mucolytic agent used in respiratory illness as well as an antidote for acetaminophen hepatotoxicity. It is recently gaining ground as a complementary therapy for cancer (Arora-Kuruganti *et al*., 1999; Chiao *et al.*, 2000; Moschou *et al*., 2008). N-Acetylcysteine has been reported to protect against testicular damage and dysfunction by the attenuation of increased testicular malondialdehyde (MDA) levels and decreased superoxide dismutase, catalase, GSH and glutathione-S-Transferase (GST) levels resulting from tetracycline-induced toxicity in rats (Farombi *et al*., 2008). It has been reported useful as adjunct antioxidant in acute lymphoblastic leukemia therapy in children (Al-Tonbary *et al.*, 2009), and in cyclophosphamide-induced hemorrhagic cystitis in rats (Jamshidzadeh *et al.*, 2009). However, its probable effect on cyclophosphamide-induced testicular toxicity is yet to be ascertained. Therefore, the aim of this study was to investigate the effects of N-acetylcysteine on sperm count, histone-protamine replacement, chromatin integrity, testicular histomorphometry and BAX Protein expression in cyclophosphamide-induced testicular toxicity in Wistar rats.

## MATERIALS AND METHODS

### Drugs and Chemicals

Cyclophosphamide (Endoxan tablet, manufactured by Baxter Oncology, Germany) and N-Acetylcysteine (Sandox SA, [Pty] Ltd) were obtained from a local pharmacy store.

### Animals

All protocols and procedures adopted in this study adhere strictly to the guidelines of the Animal Rights Committee of the University of Ibadan. Adult male rats weighing between 150g and 170g were procured from the Central Animal House, College of Medicine, University of Ibadan for use in this study. The rats were housed under a standard environmental condition in one of the animal facilities of the Central Animal House with the provision of 12h of light and 12h of darkness, and they were fed with a standard rat chow and allowed access to water and food *ad libitum* throughout the experiments.

### Experimental Groups

The animals were divided into four groups (n=5 per group). Group 1 (control) rats were orally administered with distilled water; Group 2 with cyclophosphamide (6 mg/ kg body weight/ day); Group 3 with N-Acetylcysteine (100 mg/kg body weight/day) and Group 4 with both cyclophosphamide and N-acetylcysteine at their respective dose level for twenty one days.

### Testicular histology

The testes of the rats were prefixed in Bouin-Hollande solution prior to the histologic studies using Hematoxylin and Eosin (H&E) Staining Protocol.

### Sperm Collection

The cauda epididymal region was removed and transferred to a sterile Petri dish containing 5ml of normal saline. It was thoroughly minced with a sterile scalpel to allow for spermatozoa dispersion.

### Sperm Count

For sperm counting at 1:200 dilution, the sperm samples were prepared with normal saline. For this purpose 10 µL of the sperms were added to 190 µL of saline, and then 10 µL of the diluted sperm was dropped on a Neubauer slide and we counted the average number of sperms according to Raji *et al*. (2005).

### Sperm Histone-Protamine replacement

To test for sperm histone-protamine replacement, epididymal sperm smears were made on glass slides. Each of the smears were stained with aniline blue (AB) based on the method described by Wong *et al.* (2008). Acid aniline blue is a dye that specifes between lysine-rich histone and arginine/cysteine-rich protamine; hence, it reveals the basic nuclear protein composition of spermatozoa. The sperm smears were fixed in 4% formalin solution for 5 min, rinsed in distilled water, and stained in 5% AB in 4% acetic acid (pH 3.5) solution for 5 min. The slides were then washed in distilled water, stained in 0.5% eosin for 1 min and allowed to air-dry. After which, the slides were examined at 400X magnification in a light microscope. Dark blue stained sperm heads were considered immature, characterized by nuclear histone proteins, while colorless sperm heads were considered mature sperm with protamine.

### Chromatin Condensation and DNA Integrity

Toluidine blue (TB) is a basic dye used to evaluate both sperm chromatin condensation and DNA integrity (Mello, 1982). For this staining, air-dried smears were fixed in fresh 96% ethanol-acetone (1:1) at 48ºC for 30 min, then hydrolyzed in 0.1 N HCl at 48ºC for 5 min and rinsed thrice in distilled H_2_O for 2 min. Finally, the smears were stained with 0.05% TB in McIlvain buffer, pH 3.5 (Sigma, St. Louis, MO, USA) for 10 min (Erenpreiss *et al.*, 2001). The sperm cells were assessed by light microscopy at 400X magnification, according to metachromatic staining of sperm heads in the following scores: light blue (Intact chromatin); purple (mildly abnormal chromatin); violet (severe chromatin abnormality) (Rosenborg *et al.*, 1990). The light blue spermatozoa were considered normal cells (TB^-^) and spermatozoa with violet and purple spermatozoa were considered abnormal ones (TB^+^).

### Immunohistochemistry

Five-micron thick tissue sections were deparaffinized in xylol and hydrated in a decreasing series of ethanol. Endogenous peroxidase activity was blocked by incubation in methanol containing 0.3% H_2_O_2_ for 15 min at room temperature, followed by rinsing in 0.1 M phosphate buffered saline (PBS; pH 7.4) for 5 min. The sections were then treated with citrate buffer (pH 6) for 15 min at 98ºC as antigen retrieval. Before application of specific primary antibodies, nonspecific background staining was prevented by incubation with goat serum diluted 1:10 v/v in PBS for 50 min. Then the sections incubated overnight at 4ºC with primary antibodies, including the monoclonal antibody against Bax (Mouse Monoclonal anti-Bax; sc: 7480, Santa Cruz) at 1/100 diluted in PBS containing 10% normal goat serum (NGS). After washing twice with PBS the sections were incubated with secondary antibody biotinylated anti-mouse IgG (Santa Cruz ABC Peroxidase Mouse IgG Staining Kit) at 1/100 for 50 min. Then the specimens were incubated with peroxidase-conjugated avidin biotin for 30 min at room temperature. After washing, the sections were incubated with diaminobenzidine (DAB) as chromogen, and counterstained with hematoxylin. Negative control was performed by omitting the anti-Bax antibody. Mouse thymus was used as a positive control. Two immunohistochemical slides from each animal were blindly assessed, and staining intensity was estimated using a semi-quantitative score, the H-score, as previously described by Pallares *et al.* (2005). The H-score was calculated for each section by application of the following algorithm: H-SCORE = ΣPi (i+1) (Where i is the intensity of staining (0 - no staining, 1 - weak, 2 - moderate, 3 - strong) and Pi is the percentage of stained cells for each intensity 0 to 100%).

### Data Analysis

The data was presented as mean ± SEM and analyzed using students' t- test. *p* <0.05. was considered significant.

## RESULTS

### Effects of N-Acetylcysteine on sperm count in Cyclophosphamide treated Rats

The mean sperm count is depicted on Table 1. Rats treated with cyclophosphamide only and the ones that received the cyclophosphamide and N-acetylcysteine combination, respectively, had a significant reduction in sperm; while it increased among the N-acetylcysteine-only treated rats when compared with the control animals.

**Table 1:** Effects of N-acetylcysteine on semen characteristics in cyclophosphamide treated rats.

	*Sperm count (x10^6^/ml)*
Control	115.30±8.70
CP only	69.95±7.78*
NAC only	132.20±28.71
CP + NAC	64.78±3.52*

* *P*<0.05 when compared to the control

### Effects of N-Acetylcysteine on testicular histomorphometry on Cyclophosphamide-Treated Rats

**Plates 1:**
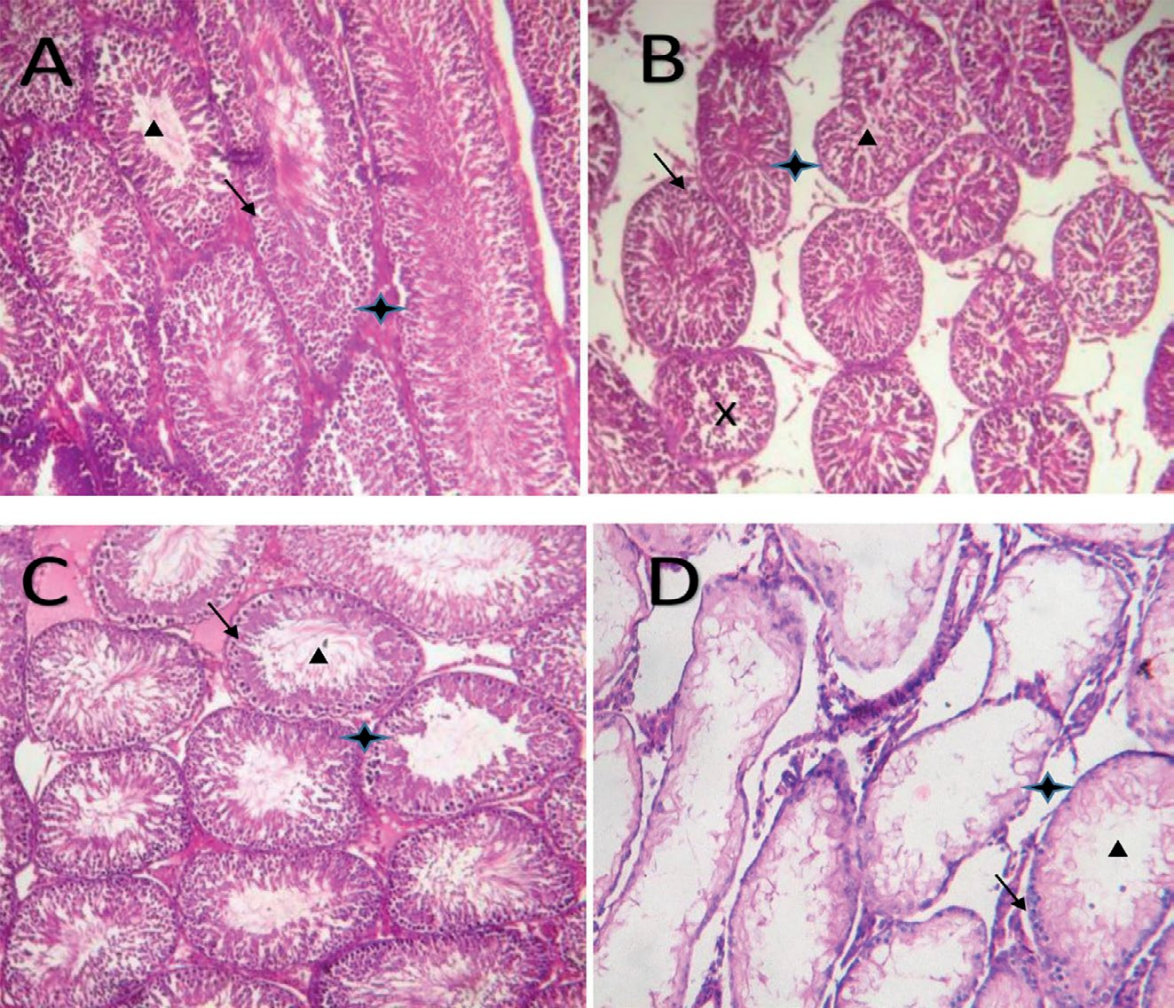
Photomicrographs of testicular tissue showing seminiferous tubules that are lined with germ cells (black arrow) at various stages of maturation to spermatozoa (black triangular box) and their respective interstitial spaces (black star box). (A) Distilled water only (B) Cyclophosphamide only (C) N-acetylcysteine only (D) Cyclophosphamide + N-acetylcysteine.

The testicular histomorphometries are shown in Plates 1 A-D. The control animals and the N-acetylcysteine-only treated rats had a normal testicular morphology with a progressive sperm development towards the lumen of the seminiferous tubules, Plate 1A and Plate 1C, respectively. However, the rats treated with cyclophosphamide only (Plate 1B), those treated with a combination of cyclophosphamide, and N-acetylcysteine (Plate 1D) showed degenerated seminiferous tubules, increased interstitial space and degenerated Leydig cells.

### Effects of N-Acetylcysteine on Sperm Maturation (Histone Protamine Replacement) in Cyclophosphamide treated rats

**Plates 2:**
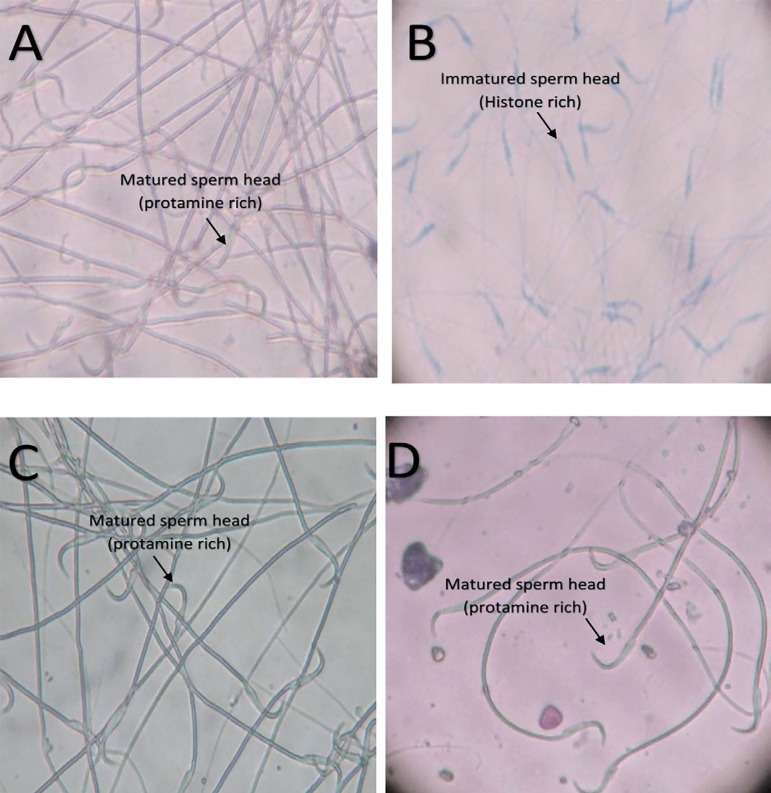
Photomicrograph showing the basic nuclear protein in spermatozoa head. (A) Distilled water only (B) Cyclophosphamide only (C) N-acetylcysteine only (D) Cyclophosphamide + N-acetylcysteine.

The histone-protamine replacement of the rats are shown in Plates 2 A-D. The results showed that except for the group 2 rats treated with cyclophosphamide only, all other groups did not take up the aniline blue stain, indicating that they had a progressive histone-protamine replacement (Plates 2A, 2C, 2D). The uptake of blue stain in group 2 rats treated with cyclophosphamide indicates that they had an impaired histone-protamine replacement with more histone retention (Plate 2B).

### Effects of N-Acetylcysteine on Chromatin Condensation (DNA Integrity) in Cyclophosphamide-treated rats

**Plates 3:**
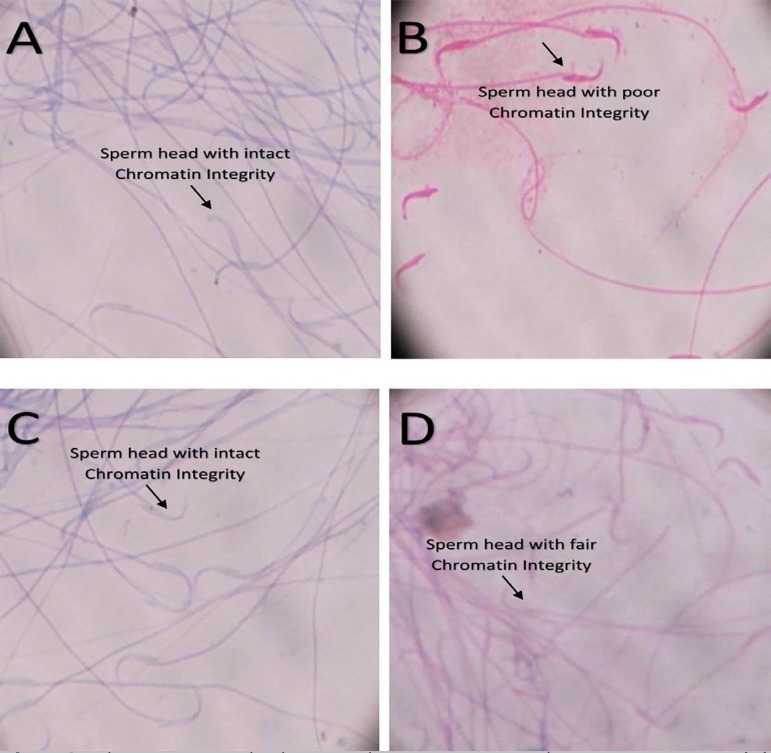
Photomicrograph showing the spermatozoa chromatin integrity. (A) Distilled water only (B) Cyclophosphamide only (C) N-acetylcysteine only (D) Cyclophosphamide + N-acetylcysteine.

Plates 3 A-D show the extent of chromatin condensation and DNA integrity in the respective groups. Normal sperm DNA integrity was found in the control group (Plate 3A) and in the N-acetylcysteine-treated group (Plate 3C). A fair DNA integrity was found in rats treated with the cyclophosphamide and N-acetylcysteine combination (Plate 3D), while a poor DNA integrity was seen in rats treated with cyclophosphamide (Plate 3B).

### Effects of N-Acetylcysteine on BAX apoptotic protein expression in Cyclophosphamide- treated rats

**Plates 4:**
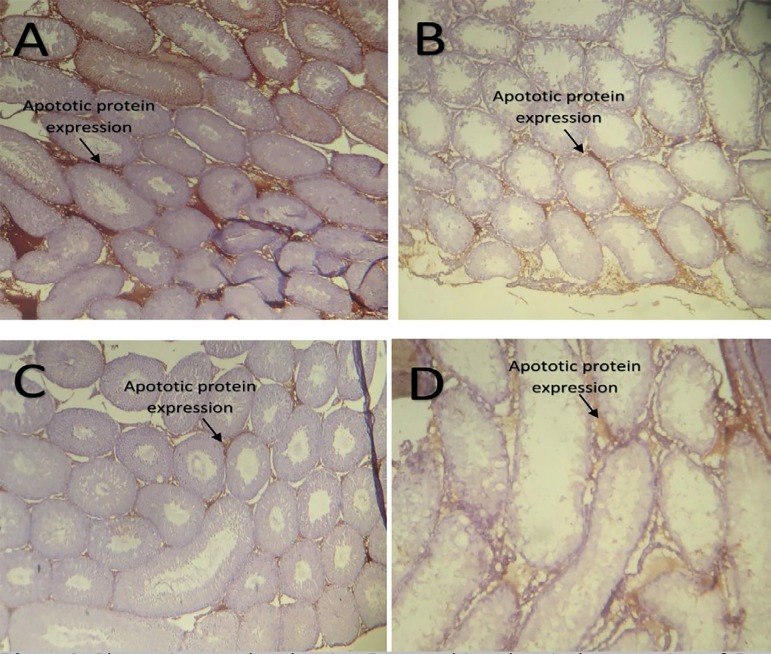
Photomicrographs showing Immunohistochemical expression of Bax apoptotic protein around the seminiferous tubule: (A) Distilled water only (B) Cyclophosphamide only (C) N-acetylcysteine only (D) Cyclophosphamide + N-acetylcysteine.

Plates 4 A-D show the expression of BAX pro-apoptotic protein in the testis. Group 1 treated with distilled water showed a very high expression in about 50% of their seminiferous tubules (Plate 4A), while group 3 rats treated with N-acetylcysteine only showed a moderate expression in about 50% of their seminiferous tubule (Plate 4C). However, a moderate expression of the BAX protein was only seen in about 20% of seminiferous tubules of group 2 rats treated with cyclophosphamide only (Plate 4B) and group 4 (Plate 4D) rats co-administered with cyclophosphamide and N-acetylcysteine.

## DISCUSSION

The aim of this study was to investigate the effects of N-acetylcysteine on sperm quality in cyclophosphamide-induced testicular toxicity. The reduction in sperm count in all animals treated with cyclophosphamide pointed to the impairing effects of cyclophosphamide on germ cell cycle in the testis. This is in line with previous reports on cyclophosphamide as an anticancer drug, with a major side effect of oligospermia in male cancer survivors (Howell *et al.*, 1999).

The degraded seminiferous tubule and Leydig cells observed in cyclophosphamide- treated rats is an evidence of its effects on testicular damage. The non-reversal of this damaged testicular seminiferous tubule in animals treated with cyclophosphamide and N-acetylcysteine indicates that N-acetylcysteine does not inhibits the primary cytotoxic role of cyclophosphamide. However, the improvement in sperm maturation in this group when compared with cyclophosphamide-treated group shows that N-acetylcysteine could play a substantial role in germ cell survival. This follows a report by Erkkilä *et al.* (1998) that N-acetylcysteine plays an important role in germ cell survival in human seminiferous tubules in an *in-vitro* study.

The histone retention seen in the cyclophosphamide-treated group is consistent with the report by Codrington *et al.* (2004; 2007) that the toxicity effects of cyclophosphamide is a stage-specific effect which is maximal during mid-spermiogenesis, a stage characterized with histone hyperacetylation and histone displacement. Matching the ameliorated histone-protamine replacement with the observed fair DNA integrity in rats treated with both Cyclophosphamide and N-acetylcysteine; N-acetylcysteine could exert part of its effects through the phosphorylated H2AX Histone - an important protein in histone-protamine replacement and DNA single strand break repair. N-acetylcysteine has been reported to enhance H2AX phosphorylation in cells exposed to doxorubicin (Kurz *et al*., 2004).

Apoptosis is a programmed cell death that is regulated at the cellular level and progress by activation of some cysteine aspartyl-specific protease (Caspases) (Häcker, 2000). It is characterized by membrane blebbing, cell volume shrinkage, chromatin condensation, cytoplasmic vacuolization and disassembly of the cell into membrane-bound remnants, termed apoptotic bodies (Majno & Joris, 1995) that are eventually picked up by phagocytic cells. The initiation of caspase activation may happen either via the mitochondria (intrinsic), or through a cell-death receptor (extrinsic) (Igney & Krammer, 2002), Granzyme (Martinvalet *et al.,* 2005), or via the endoplasmic reticulum (Szegezdi *et al.,* 2003); however, the primary effector of chemotherapy-induced apoptosis is the mitochondria pathway (Debatin *et al.,* 2002). In the mitochondria pathway, the intracellular susceptibility to apoptosis depends on the signal received from a damaged DNA component and the interaction between pro-apoptotic protein, majorly BAX and other anti-apoptotic protein of the Bcl-2 family (Farrow & Brown, 1996).

The altered DNA integrity observed in animals treated with cyclophosphamide in this study indicates the vulnerability of their germ cells to apoptosis. This is consistent with the report from Basu & Haldar (1998) that DNA damage is one of the signals for the progression of apoptosis. The reduced BAX expression seen in the animals treated with cyclophosphamide in this study is also consistent with earlier reports of reduced BAX expression following the exposure of prostate (Haldar *et al*., 1996) or breast (Srivastava *et al*., 1998) cancer cells to Taxol - a chemotherapeutic agent. Matching the expression of BAX protein in these groups treated with cyclophosphamide with their altered chromatin integrity and degraded seminiferous tubules may hint that apoptosis have occurred in a pathway independent of Bax protein. This is in line with the results from Russell *et al.* (2002), who reported the occurrence of testicular atrophy and degeneration of germ cells in BAX-deficient mice. It has also been documented in an *in-vitro* study by Krajewski *et al.* (1996) that for most anticancer drugs, elevation of the Bcl-2/BAX ratio due to changes in the expression of any of the proteins do not prevent drug-induced apoptosis.

On the other hand, a normal testicular morphometry and intact chromatin matched with high BAX expression observed in the control and N-acetylcysteine-treated groups might probably be due to the regulatory role of BAX proteins in germ cell survival (Rodriguez *et al.*, 1997; Rucker *et al.*, 2000; Russell *et al.*, 2002).

## CONCLUSION

N-acetylcysteine may improve sperm histone-protamine replacement, which is one of the stage-specific effects of cyclophosphamide toxicity during spermatogenesis. Understanding the role of N-acetylcysteine in the amelioration of sperm DNA damage, particularly in the repair of DNA single stand breakage in cyclophosphamide toxicity might prove it a promising adjunct drug.
